# Radiofrequency vs. Cryoballoon vs. Thoracoscopic Surgical Ablation for Atrial Fibrillation: A Single-Center Experience

**DOI:** 10.3390/medicina57101023

**Published:** 2021-09-26

**Authors:** Hee-Jin Kwon, Ji Hoon Choi, Hye Ree Kim, Seung-Jung Park, Dong Seop Jeong, Young Keun On, June Soo Kim, Kyoung-Min Park

**Affiliations:** 1 Division of Cardiology, Department of Internal Medicine, Heart Vascular and Stroke Institute, Samsung Medical Center, Sungkyunkwan University School of Medicine, Seoul 06351, Korea; subakhj@gmail.com (H.-J.K.); ji.hoon.choi@samsung.com (J.H.C.); hrmanse@naver.com (H.R.K.); orthovics@gmail.com (S.-J.P.); oykmd123@gmail.com (Y.K.O.); juneskim@skku.edu (J.S.K.); 2 Department of Thoracic and Cardiovascular Surgery, Heart Vascular and Stroke Institute, Samsung Medical Center, Sungkyunkwan University School of Medicine, Seoul 06351, Korea; cabg@korea.com

**Keywords:** atrial fibrillation, radiofrequency catheter ablation, cryoballoon ablation, thoracoscopic surgical ablation

## Abstract

*Background and Objectives*: Cryoballoon ablation (CBA) and totally thoracoscopic surgical ablation (TTA) have emerged as alternatives to radiofrequency catheter ablation (RFCA) for atrial fibrillation. In this study, we describe our experience comparing patient characteristics and outcomes of RFCA, CBA, and TTA. *Materials and Methods*: We retrospectively analyzed data from patients who underwent RFCA, CBA, or TTA. Both atrial fibrillation (AF)- and atrial tachyarrhythmia (ATa)-free survival rates were compared using time to recurrence after a 3-month blanking period (defined by a duration of more than 30 s). All patients were regularly followed using 12-lead ECGs or Holter ECG monitoring. *Results:* Of 354 patients in this study, 125 underwent RFCA, 97 underwent CBA and 131 underwent TTA. The TTA group had more patients with persistent AF, a larger LA diameter, and a history of stroke. The CBA group showed the shortest procedure time (*p* < 0.001). The CBA group showed significantly lower AF-free survival at 12 months than the RFCA and TTA groups (RFCA 84%, CBA 74% and TTA 85%, *p* = 0.071; *p* = 0.859 for TTA vs. RFCA, *p* = 0.038 for RFCA vs. CBA and *p* = 0.046 for TTA vs. CBA). There were no significant differences in ATa-free survival among the three groups (*p* = 0.270). There were no procedure-related adverse events in the RFCA group, but some complications occurred in the CBA group and the TTA group (6% and 5%, respectively). *Conclusions*: RFCA and CBA are effective and safe as first-line treatments for paroxysmal and persistent AF. In some high-risk stroke patients, TTA may be a viable option. It is important to consider patient characteristics when selecting an ablation method for AF.

## 1. Introduction

Atrial fibrillation (AF) is the most common cardiac arrhythmia in adults. The prevalence of AF increases with age and occurs mostly in the elderly. However, it can also occur in young adults, adolescence, or childhood and may be precipitated by hypertension, hyperthyroidism, or lifestyle factors such as endurance sport, alcohol consumption, or even smoking and channelopathies [1,2,3,4,5,6]. AF decreases quality of life and is associated with deleterious outcomes such as heart failure, ischemic stroke, and death [7,8]. The standard treatment for AF that occurs even after correcting modifiable precipitating factors is catheter ablation. Catheter ablation has been shown in many studies to be superior to anti-arrhythmic drugs (AADs) for the maintenance of sinus rhythm and symptom improvement [9,10]. In the most recent medical guidelines, catheter ablation is recommended as a first-line treatment for some patients [11]. However, recurrences of arrhythmia are still common, and there have been many advances in creating durable transmural lesion sets. Of these, cryoballoon ablation (CBA) and thoracoscopic surgical ablation (totally thoracoscopic ablation, TTA) have been developed as alternatives to radiofrequency catheter ablation (RFCA).

In previous studies, CBA was shown to be as effective and safe as RFCA [12]. Since the encircling lesion of the pulmonary vein (PV) antrum is created with a single energy application, the procedure time is shorter [12]. In addition, based on characterized cryothermal lesions that preserve tissue architecture, CBA has a lower risk of cardiac perforation or thrombogenicity [13]. TTA is based on the Cox-maze operation and is a minimally invasive approach. TTA is thought to be more effective than RFCA because it directly creates homogeneous transmural lesions. Substrate modification and LAA exclusion can be performed simultaneously. Previous studies showed that TTA had better effects than RF, but the risk of complications was also high.

There have been many studies comparing TTA and CBA to RFCA, but there are no studies comparing all three simultaneously. In addition, due to the differences in enrolled patient characteristics, procedures, or follow-up methods in each study, outcomes were highly variable, and direct comparison between studies is difficult. Therefore, in this study, we present our experience comparing patient characteristics and outcomes among RFCA, CBA, and TTA.

## 2. Materials and Methods

### 2.1. Study Population

**Figure 1:**
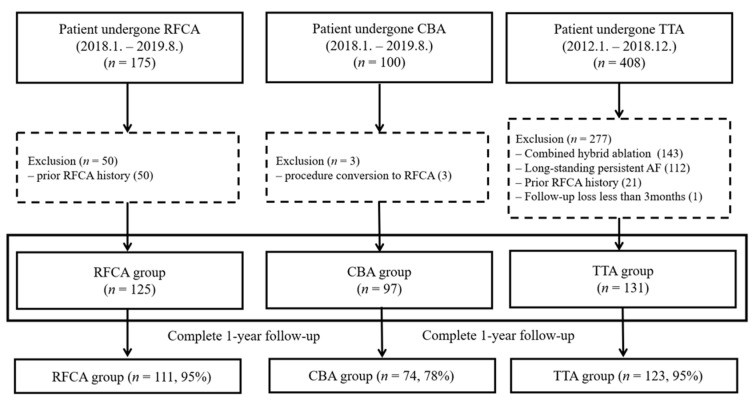
Study population.

We retrospectively enrolled all consecutive AF patients who underwent RFCA or CBA between January 2018 and August 2019 and those who underwent TTA between January 2012 and December 2018. Patients aged >18 years with symptomatic paroxysmal or persistent AF refractory to AADs who underwent a first ablation procedure were retrospectively enrolled in this study. Patients with long-standing persistent AF, a history of prior catheter or surgical ablation for AF, procedure conversion from CBA to RFCA or from TTA to RFCA due to incomplete procedures, or a follow-up period of less than three months were excluded (Figure 1). Demographic, procedural, and follow-up data were collected through a careful review of electronic medical records. Procedure methods were selected by the operator based on the patient’s condition and preference as well as the operator’s preference and experience. In particular, TTA was selectively performed in patients with paroxysmal or persistent AF who had a history of stroke, large LA diameter, a history of ASD device closure, or if the patient preferred surgical treatment. The type of AF, recurrence, and complications were defined in accordance with current guidelines [11,14]. AADs were discontinued for five half-lives before the study. All patients were prescribed anticoagulants for at least four weeks before the procedure. This study was approved by the Institutional Review Board of Samsung Medical Center and the need for informed consent was waived (IRB No. 2020-06-159).

### 2.2. Catheter Ablation: Radiofrequency and Cryoballoon

We routinely performed transesophageal echocardiography to rule out intracardiac thrombus and cardiac computed tomography (CT) for structural evaluation prior to the procedure. All procedures were performed under general anesthesia with a femoral approach. Trans-septal puncture was performed using fluoroscopy, and blood pressure was monitored during the procedure. Systemic anticoagulation with intravenous heparin was initiated immediately after the transseptal insertion of sheaths, and activated clotting time (ACT) was checked every 30 min for a target ACT level of about 300 s. Esophageal temperature was monitored in all patients during the procedure. All procedures were performed by two experienced electrophysiologists (YKO, KMP).

#### 2.2.1. Catheter Ablation with Radiofrequency

**Figure 2:**
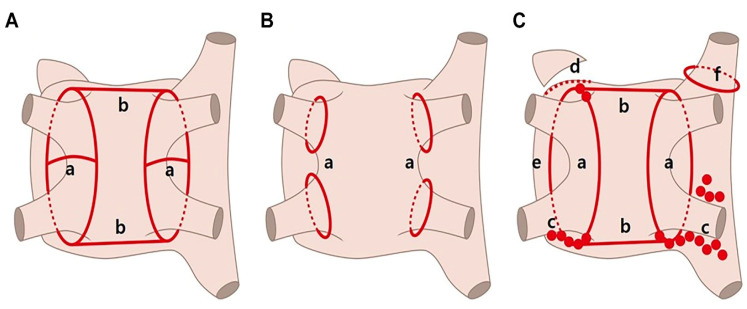
Schematic map of the three procedures. (**A**) Radiofrequency catheter ablation. (**B**) Cryoballoon ablation. (**C**) Totally thoracoscopic ablation. PV, pulmonary vein; GP, ganglionated plexus; LA, left atrium; SVC, superior vena cava. a, PV isolation. b, Roof and posterior line ablation; c, GP ablation; d, LA auricle resection; e, Division of Marshall vein; f, SVC ablation.

During catheter ablation with radiofrequency, a 3.5 mm tip contact-force (CF)-sensing irrigated tip ablation catheter (Thermocool Smartouch, Biosense Webster Inc., Diamond bar, CA, USA) was used with a three-dimensional mapping system (CARTO3, Biosense Webster Inc.). We assessed the PV potential using a circulator mapping catheter (LASSO NAV, Biosense Webster) and ablated the antrum of the PV point by point. The targeted contact force was 10 to 40 g. The energy was delivered under power control with a pre-set power of 30 watts (25 W, especially in the posterior wall). Ablation tags were automatically displayed with the VisiTag module. The predefined parameters were catheter stability with range of motion ≤2 mm during >4 s and minimum force ≥10 g over 70% of the time. If any of these criteria were not met, tags were annotated at the operator’s command when the RF duration was 20 s (reduced to 15 s at the posterior wall). PV isolation was mandatory for all patients while additional roof/posterior line ablation was performed at the operator’s discretion (Figure 2A). After ablation, we confirmed the entrance block of all four PVs and bidirectional block of linear lines. Non-PV triggers were confirmed by infusion of high-dose isoproterenol and rapid atrial pacing.

#### 2.2.2. Cryoballoon Catheter Ablation 

LA access was obtained via femoral venous access and a trans-septal approach. A dedicated 15 Fr delivery sheath (FlexCath Advance, Medtronic, Inc., Minneapolis, MN, USA) was introduced into the LA over the wire along with the cryoballoon catheter (Arctic Front Advance, Medtronic). A dedicated inner lumen mapping catheter (Achieve, Medtronic) was commonly used for delivery of the cryoballoon and sheath. The Achieve mapping catheter/guidewire was maneuvered into a pulmonary vein. The inflated cryoballoon was advanced toward the antral surface of the PV and cryoapplication was initiated. PV isolation was confirmed using a spiral mapping catheter, and when the temperature dropped from −40 °C to −60 °C and time to isolation (TTI) was achieved within 60 s, cryoablation was applied for 180 s. If the temperature did not reach an acceptable level or PVI was not achieved within 60 s, the cryoablation lesion was interrupted and additional applications were performed after rewarming and repositioning the balloon catheter (Figure 2B). Phrenic nerve pacing was conducted using a diagnostic catheter at the level of the right subclavian vein during right-sided ablation and diaphragmatic movement was monitored. Catheter ablation was immediately terminated upon weakened diaphragmatic response. 

### 2.3. Thoracoscopic Surgical Ablation

TTA refers to a video-assisted thoracoscopic surgical ablation technique performed without the assistance of the Da Vinci system (Intuitive Surgical, Sunnyvale, CA, USA) or cardiopulmonary bypass. This technique involves a bilateral approach requiring only three holes per side: a 5 mm port introduced in the fourth intercostal space at the midaxillary line and two ports placed in the third intercostal space at the anterior axillary line and in the sixth intercostal space at the midaxillary line. After pericardial tenting, a lighted dissector (AtriCure LumitipDissector, Atricure, Inc., Cincinnati, OH, USA) was used to pass a rubber band under the PV antrum through the oblique sinus. An AtriCure Isolator Transpolar Clamp was positioned around the PV antrum through connection to the rubber band. Bipolar radiofrequency energy was delivered six times to the clamps for PV isolation. Superior and inferior lines connecting both PV isolation lines were created using a linear pen device (Atricure, Inc.). The above method was first performed on the right side and repeated on the left side. Ganglionated plexuses were subsequently ablated with bipolar energy under high-frequency pacing. After PV and ganglionated plexus ablation, the ligament of Marshall was dissected and ablated. Once ablations were completed and conduction block was confirmed, the left atrial auricle was removed with an endoscopic stapling device (Figure 2C). All operations were performed by one experienced surgeon (DSJ).

### 2.4. Post-Procedural Care and Follow-Up 

Patients who underwent RFCA or CBA started taking oral anticoagulants when hemostasis was confirmed at the femoral puncture site approximately 6 h after the procedure. The absence of pericardial effusion was confirmed 4 h after the surgery in patients who underwent TTA, and heparin infusion was started, targeting an activated partial thromboplastin time of 60–80 s and switching to oral anticoagulants the day after surgery. All patients continued to take antiarrhythmic drugs unless contraindicated.

Patients underwent follow-up 1 month, 3 months, 6 months, and 12 months post-procedure. At every visit, 12-lead ECG or 24-hour ambulatory ECG monitoring was performed along with evaluation of any symptoms of AF recurrence. After a 3-month blanking period, recurrence of atrial tachyarrhythmia (ATa) was defined as when AF, flutter, or atrial tachycardia was detected for more than 30 s. The AF-free survival rate was defined separately and only included recurrences of AF. If the sinus rhythm was maintained stably during follow-up, AADs were discontinued at 3 months or up to 6 months. Antiarrhythmic drugs were continued if atrial arrhythmia recurred or if the patient complained of symptoms even without documentation on ECG. Procedure-related complications were defined according to the 2017 HRS/EHRA/ECAS/APHRS/SOLACE expert consensus statement on catheter and surgical ablation of AF ablation [14]. Major adverse cardiovascular and cerebral events (MACCEs) were also collected in all patients during the follow-up period.

### 2.5. Statistical Analysis

Data were analyzed using commercial software (SPSS version 22.0, IBM Co., Chicago, IL, USA). Continuous variables are presented as the mean ± standard deviation (SD) or median with interquartile range (IQR) depending on the distribution. Categorical variables are described as numbers and percentages. Analysis of variance (ANOVA) testing was performed for statistical comparison between nominal measures, and chi-square test was performed for categorical data. Independent K-sample test was performed for nonparametric data. Kaplan-Meier curve with log-rank tests was used to compare differences in the arrhythmia-free survival rate between groups. Cox regression analysis was used to identify predictors of AF recurrence. A two-tailed *p*-value of <0.05 was considered statistically significant.

## 3. Results

### 3.1. Baseline Characteristics 

Of the 353 patients who were treated with AF ablation, 125 patients underwent RF ablation (RFCA group), 97 patients underwent CBA (CBA group), and 131 patients underwent TTA (TTA group). The mean age was 57 ± 10 years and 83% of patients were male. The TTA group had more patients with persistent AF (*n* = 91, 74%, *p* < 0.001). Prior stroke was more likely to be in the TTA group (RFCA vs. CBA vs. TTA, 7% vs. 6% vs. 28%, *p* < 0.001). The mean CHAD2S2-VASc score was 1.3 ± 1.3, and 128 patients (36%) scored higher than 2 points. In the TTA group, the mean LA diameter was larger than that of the other groups (RFCA vs. CBA vs. TTA, 41.4 ± 5.8 mm vs. 41.7 ± 6.3 mm vs. 46.2 ± 6.4 mm, *p* < 0.001). At the 6-month follow-up, the use of AAD was more frequent in the TTA group. The baseline characteristics among the 3 groups are shown in Table 1.

### 3.2. Procedural Characteristics 

The procedure time was shortest in the CBA group at 90 ± 20 min, followed by statistically significant increases in the RFCA and TTA groups (139 ± 34 and 157 ± 39 min, respectively, *p* < 0.001). However, the CBA group also had a significantly longer fluoroscopy time than the RFCA group (RFCA vs. CBA, 25 ± 11 vs. 29 ± 10 min). The TTA group had a greater mean length of hospital stay than the other groups. PV isolation was mandatory in all patients. Additional line ablation such as roof line, posterior line, and SVC isolation were performed more frequently in the TTA group than in the RFCA group. In addition, ganglionic plexus ablation was performed in 90% and LAA exclusion was performed in 98% of the TTA group. Except for 8% of patients who underwent CTI ablation, no patients underwent additional ablation in the CBA group. Procedural details are shown in Table 2.

### 3.3. Follow-Up

The overall mean follow-up duration was 23 ± 18 months, and the mean follow-up period of each group was 18 ± 5, 15 ± 6 and 40 ± 21 months in the RFCA, CBA, and TTA groups, respectively.

**Figure 3:**
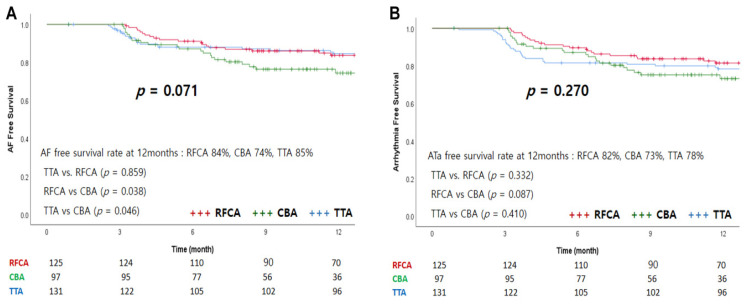
Event-free survival curves of the three groups. (**A**) AF-free survival curve, (**B**) Arrhythmia-free survival curve.

In total, 111 (95%), 74 (78%), and 123 (95%) patients in each group completed follow-up of more than 1 year. At 12 months, the AF-free survival rates for RFCA, CBA, and TTA were 84%, 74%, and 85%, respectively (*p* = 0.071; *p* = 0.859 for TTA vs. RFCA, *p* = 0.038 for RFCA vs. CBA, and *p* = 0.046 for TTA vs. CBA; Figure 3A). The ATa-free survival rate was 82% for the RFCA group, 73% for the CBA group, and 78% for the TTA group (*p* = 0.270; *p* = 0.332 for TTA vs. RFCA, *p* = 0.087 for RFCA vs. CBA, *p* = 0.410 for TTA vs. CBA; Figure 3B). The recurrent types of ATa are shown in Table 3. One patient in the RFCA group recurred with AF and atrial flutter. In the TTA group, one patient recurred with typical atrial flutter with AF, and the other one patient had recurred AF and atrial tachycardia. 

**Figure 4:**
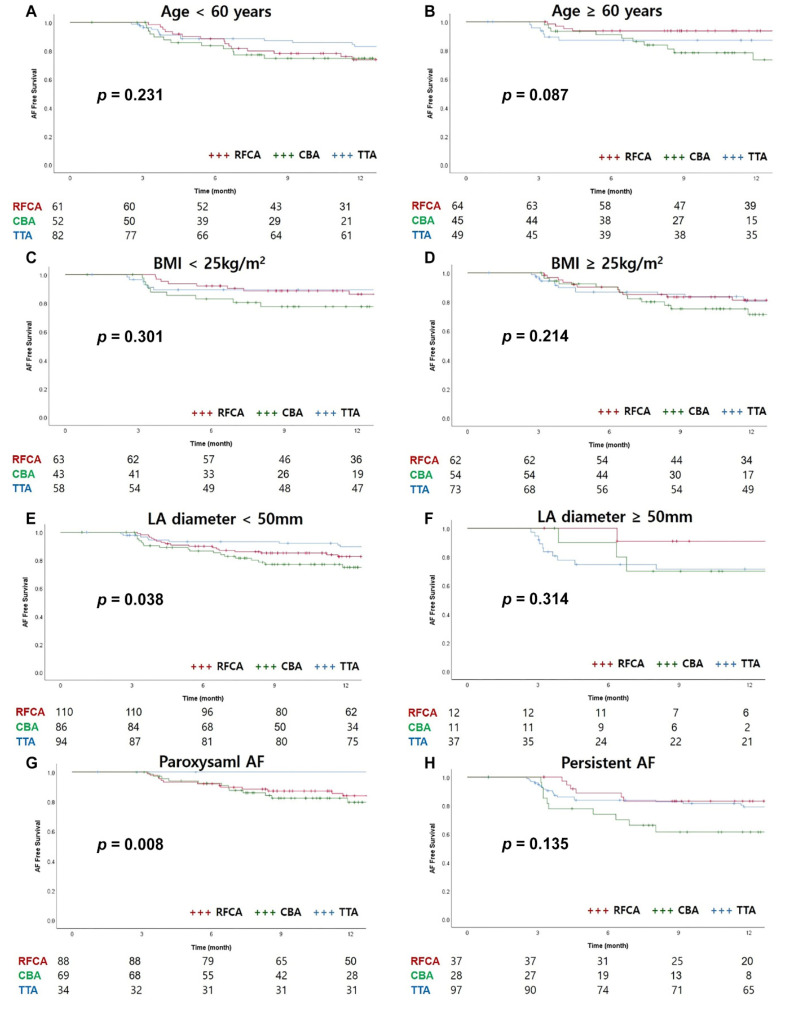
Subgroup analysis of AF-free survival curves. (**A**) Age < 60 years, (**B**) Age ≥ 60 years, (**C**) BMI < 25 kg/m^2^, (**D**) BMI ≥ 25 kg/m^2^, (**E**) LA diameter < 50 mm, (**F**) LA diameter ≥ 50 mm, (**G**) Paroxysmal AF, (**H**) Persistent AF, body mass index; LA, left atrium; AF, atrial fibrillation.

In the subgroup analysis of the AF-free survival rate, considering age, BMI, or LA diameter, there were no significant differences among the three groups (Figure 4). The AF-free survival rate in paroxysmal AF was significantly higher in the TTA group than in the other groups (*p* = 0.008). However, in persistent AF, the CBA group tended to have a lower AF-free survival rate, but there was no statistically significant difference (*p* = 0.315) (Figure 4G,H). 

In univariate analysis, persistent AF, LA diameter, and BMI were associated with AF recurrence. In multivariate analysis, only LA diameter was associated with AF recurrence. Analyzing the risk among ablation methods, there was no difference in the univariate analysis. However, after adjusting for age, sex, BMI, and LA diameter, the CBA group had a higher risk of recurrence than the other groups (HR 1.773, CI 1.029–3.055, *p* = 0.339) (Table 4).

### 3.4. Procedure-Related Complications

A total of 12 adverse events (3.4%) were reported in all patients. There was no difference in the overall incidence of procedure-related complications between groups, with three cases (2%) in the RFCA group, four cases (4%) in the CBA group, and five cases (4%) in the TTA group (*p* = 0.739, Table 5). However, there were only two severe adverse events; one death and one stroke occurred in the TTA group. One patient in the TTA group died due to cardiac arrest of unknown cause during hospitalization due to mild pericarditis. Another patient who discontinued anticoagulant therapy immediately after surgery experienced an embolic stroke four days post-operative. There were no atrial–esophageal fistulas or cases of cardiac tamponade. One patient in the CBA group had a small pericardial effusion after the procedure, but this improved without further intervention. Two patients suffered transient phrenic nerve injuries from which both recovered before discharge. All patients who underwent pacemaker implantation (two patients in the CBA group and two patients in the TTA group) experienced sinus node dysfunction before the procedure. During the follow-up period, one patient in the TTA group experienced an embolic stroke. 

## 4. Discussion

In this study, we compared the safety and efficacy of different AF ablation techniques through a single-center experience. A main finding of this study was that the CBA group showed significantly lower AF-free survival at 12 months compared to the RFCA or TTA groups. A second main finding was that there was no significant difference in the ATa-free survival rate among the three groups in patients with paroxysmal or persistent AF. Most CBA patients received PV isolation only, and this group had the shortest procedure time. The TTA group was associated with the longest hospitalization period. There were no procedure-related adverse events in the RFCA group, but some complications occurred in the CBA group and the TTA group.

Since the FIRE and ICE study proved that CBA is not inferior to RFCA in terms of efficacy and safety, CBA has become one of the most widely-used AF treatment options [12]. A recent study using next-generation ablation technologies also showed similar efficacy between CBA and RFCA [15]. This research group commented that the ATa-free survival rate at 12 months was 53%, which was lower than other studies, because of continuous cardiac rhythm monitoring; but the AF burden was reduced by more than 98%, demonstrating that CBA and RCFA were sufficiently effective treatment options for AF. Even in a recent study comparing RFCA using high-power short-term ablation and CBA, CBA was effective and the treatment time was shorter [16]. In our study, the AF-free survival rate was significantly higher in the RFCA and TTA groups than in the CBA group. ATa-free survival results were consistent with previous studies; a similar efficacy was seen between the CBA and RFCA groups and between the CBA and TTA groups. However, after adjusting for LA diameter, CBA showed a higher association with recurrence than TTA. Whether left atrial enlargement is the cause or result of atrial fibrillation remains unclear, but LA enlargement is associated with AF recurrence. Structural remodeling, atrial hypertrophy, and stretch can lead to alterations in ionic currents and electrical remodeling. Cardiac endothelin-1 (ET-1) expression responds to wall stress, can promote myocyte hypertrophy and interstitial fibrosis, and correlates with enlarged LA size [17]. Since our study included patients with relatively large atrial diameters, especially in the TTA group, the risk of recurrence was higher in the CBA group in which additional ablation other than PV isolation was not possible. 

TTA theoretically creates a homogeneous and constant lesion by direct application and has advantages in substrate medication. In addition, LAA exclusion can be performed simultaneously to prevent stroke. However, TTA is invasive and has a high risk of complications. Early studies comparing surgical ablation and catheter ablation showed significantly superior outcomes in surgical ablation. In the FAST study, surgical ablation was found to have better AF-free survival at 12 months than catheter ablation (65.6% vs. 36.5%, *p* = 0.002) [18]. De Matt et al. reported that surgical ablation was more effective in patients with AF who were without prior ablations [19]. However, in the former study, two-thirds of the enrolled patients had a prior failed catheter ablation. Additionally, in both studies, catheter ablation had a lower AF-free survival rate than that demonstrated in recent studies using the old ablation technique. In recent studies, although in long-standing persistent AF, TTA showed a similar success rate to RFCA. In addition, RFCA provided greater symptom improvement during follow-up than TTA [20]. Furthermore, the incidence of complications was significantly lower than that of the initial study, showing no statistically significant difference from RFCA. However, serious complications tended to occur were similar to previous studies. There were no complications in the RFCA group; the risk was significantly lower in this group than in the other groups. There was a similar risk of complications between the CBA and TTA groups. Although all patients underwent LAA resection, one patient with previous strokes had stroke complications in the TTA group.

Radiation exposure related to transcatheter ablation carries small but non-negligible stochastic and deterministic effects on health [21,22]. With the development of tools such as 3D mapping systems and intracardiac echocardiography, the current trend is changing towards a non-fluoroscopic approach [23,24,25]. In this study, RFCA and CBA showed fluoroscopy times of 25 and 29 min, respectively. TTA had no X-ray exposure, and there was a longer X-ray exposure in the CBA group. Non-fluoroscopic ablation reduced the use of X-rays without compromising the duration, effectiveness, and safety of the procedure. Also, although CBA requires more extensive radiation guidance than RFCA, ways to reduce radiation exposure are being explored in CBA as well [26].

Although there are differences in the patient populations, our study also showed similar results to recent studies. Current guidelines recommend TTA for symptomatic paroxysmal or persistent AF when a patient has failed catheter ablation or for persistent AF with risk factors for recurrence [11]. As described earlier, our center has adhered to a treatment strategy that includes the performance of TTA as the first ablation method only for selected paroxysmal or persistent AF patients with a history of stroke, larger LA diameter, a history of ASD device closure, or a preference for TTA. Our study provides additional evidence that RFCA and CBA are sufficient first-line treatments in paroxysmal/persistent AF patients without a history of previous stroke. Also, this study suggests that TTA is a preferred first-line option in patients with a history of recurrent strokes even when AF is paroxysmal or persistent. To the best of our knowledge, this is the first single-center study to compare these three procedural options for persistent/paroxysmal AF.

This study had several limitations. First, it was a retrospective and single-center observational study, so it was difficult to compare the outcomes of each ablation method head-to-head in this study. However, since each ablation procedure was performed at a single center with the updated technique at the time, there would have been less bias in drawing conclusions from the outcomes of each procedure. Additionally, one 3D mapping system (CARTO3, Biosense Webster Inc.) was used in all RFCA procedures. The other systems with specific tools for AF ablation (ABBOTT and BOSTON SCIENTIFIC) may lead to different procedural and clinical outcomes. Even though we carefully reviewed patient medical records, it was not uncommon for data to be insufficient or censored during follow-up. In particular, minor complications may have been underreported. Since ECG was performed at 3–6-month intervals and patients had intermittent rather than continuous monitoring, AF recurrence, including asymptomatic AF, may be underestimated. However, since all patients were followed with ECG or 24-h Holter monitoring, an impact on comparisons among the three groups is less likely. 

## 5. Conclusions

In this study, we compared the results of RFCA, CBA, and TTA as viable treatments for AF. Although there were no significant differences in efficacy among the three methods, the procedure time was shorter in CBA and RFCA. RFCA and CBA are effective and safe as first-line treatments for paroxysmal and persistent AF. In some high-risk stroke patients, TTA may be a viable option. It is important to consider patient characteristics when selecting an ablation method for AF, and large-scale follow-up studies are needed.

## Figures and Tables

**Table 1:** Baseline characteristics.

	Total (*n* = 353)	RFCA (*n* = 125)	CBA (*n* = 97)	TTA (*n* = 131)	*p*-Value
Gender (Female)	60 (17%)	22 (18%)	21 (22%)	17 (13%)	0.222
Age (years)	56.9 ± 9.8	56.9 ± 10.5	57.3 ± 9.7	56.6 ± 9.2	0.875
Body mass index	25.5 ± 3.1	25.1 ± 2.2	25.6 ± 3.2	25.8 ± 3.7	0.156
Comorbidities					
Hypertension	140 (40%)	53 (42%)	43 (44%)	45 (34%)	0.256
Diabetes mellitus	41 (12%)	18 (14%)	9 (9%)	14 (11%)	0.471
Prior stroke/TIA	52 (15%)	9 (7%)	7 (6%)	36 (28%)	<0.001
Heart failure	18 (5%)	4 (3%)	4 (4%)	10 (8%)	0.234
C-V score	1 (0–2)	1 (0–2)	1 (0–2)	1 (0–2)	0.346
C-V score ≥2	128 (36%)	40 (32%)	31 (32%)	57 (44%)	0.088
Type of AF					<0.001
Paroxysmal	191 (54%)	89 (71%)	69 (71%)	34 (26%)	
Persistent	162 (46%)	37 (29%)	28 (29%)	91 (74%)	
Echocardiographic parameter					
LV EF (%)	61.5 ± 7.3	62.3 ± 6.2	60.9 ± 8.5	61.2 ± 7.2	0.286
LA diameter (mm)	43.3 ± 6.5	41.4 ± 5.8	41.7 ± 6.3	46.2 ± 6.4	<0.001
LA volume index	43.3 ± 14.9	40.1 ± 13.3	40.6 ± 12.9	48.4 ± 16.3	<0.001
LA > 50 mm	60 (17%)	12 (10%)	11 (11%)	37 (28%)	<0.001
Medication					
AAD * at discharge	301 (85%)	108 (86%)	83 (86%)	110 (84%)	0.856
AAD * at 6 months	164 (47%)	50 (40%)	43 (44%)	75 (57%)	0.017

C-V score represented median with interquartile range. * AAD include class I or III; AAD, antiarrhythmic drugs; RFCA, radiofrequency catheter ablation; CBA, cryoballoon ablation; TTA, totally thoracoscopic ablation; TIA, transient ischemic attack; C-V, CHA_2_DS_2_-VASc, congestive heart failure, high blood pressure, Age 75, Diabetes, previous Stroke, Vascular disease, Age 65–74, Sex; AF, atrial fibrillation; LV, left ventricle; EF, ejection fraction; LA, left atrium.

**Table 2:** Procedural characteristics.

	RFCA (*n* = 125)	CBA (*n* = 97)	TTA (*n* = 131)	*p*-Value
Procedure duration (min)	139 ± 34	90 ± 20	157 ± 39	<0.001
Fluoroscopy time (min)	25 ± 11	29 ± 10	-	0.010
Length of hospital stay (days)	3.7 ± 0.6	3.8 ± 0.9	10.2 ± 4.7	<0.001
Pulmonary vein isolation	125 (100%)	97 (100%)	131 (100%)	
Roof line	51 (42%)	0 (0%)	120 (92%)	
Posterior line	46 (37%)	0 (0%)	129 (99%)	
Mitral isthmus line	33 (32%)	0 (0%)	0 (0%)	
Endocardial CS	17 (17%)	0 (0%)	0 (0%)	
SVC isolation	18 (18%)	0 (0%)	52 (40%)	
CTI line	84 (68%)	7 (8%)	0 (0%)	
Ganglionic plexi	0 (0%)	0 (0%)	118 (90%)	
LAA exclusion	0 (0%)	0 (0%)	128 (98%)	

RFCA, radiofrequency catheter ablation; CBA, cryoballoon ablation; TTA, totally thoracoscopic ablation; CS, coronary sinus; SVC, superior vena cava; CTI, cavotricuspid isthmus; LAA, left atrial appendage.

**Table 3:** The recurrence types of atrial tachyarrhythmias.

	RFCA (*n* = 125)	CBA (*n* = 97)	TTA (*n* = 131)
Any atrial tachyarrhythmia	24 (19%)	25 (26%)	41 (31%)
Atrial fibrillation	20 (16%)	24 (25%)	29 (22%)
Atrial flutter	5 (4%)	1 (1%)	13 (10%)
Atrial tachycardia	0 (0%)	0 (0%)	1 (1%)

RFCA, radiofrequency catheter ablation; CBA, cryoballoon ablation; TTA, totally thoracoscopic ablation.

**Table 4:** Cox regression for predictors of recurrence.

	Univariate Analysis		Multivariate Analysis *	
	HR (95% CI)	*p*-Value	HR (95% CI)	*p*-Value
Age	0.998 (0.978–1.109)	0.868	0.993 (0.970–1.017)	0.993
Gender (Female)	0.873 (0.495–1.542)	0.641	0.900 (0501–1.617)	0.725
BMI	1.099 (1.038–1.164)	0.001	1.054 (0990–1.121)	0.098
Hypertension	1.230 (0.815–1.855)	0.324	-	
LA diameter	1.073 (1.039–1.108)	<0.001	1.082 (1.046–1.119)	<0.001
Type of AF			-	
Paroxysmal	Reference			
Persistent	1.942 (1.279–2.947)	0.002		
Ablation method				
TTA	Reference		Reference	
RFCA	0.854 (0.511–1.430)	0.273	1.333 (0.722–2.301)	0.302
CRYO	1.289 (0.765–2.171)	0.341	1.773 (1.029–3.055)	0.039

* Age, sex, BMI, LA diameter, and ablation method were included in multivariable analysis. HR, hazard ratio; CI, confidence interval; BMI, body mass index; LA, left atrium; AF, atrial fibrillation; RFCA, radiofrequency catheter ablation; CBA, cryoballoon ablation; TTA, totally thoracoscopic ablation.

**Table 5:** Procedure-related complications.

	RFCA (*n* = 125)	CBA (*n* = 97)	TTA (*n* = 131)
Procedural complication	3 (2%)	4 (4%)	5 (4%)
Death	0 (0%)	0 (0%)	1 (1%)
Stroke/TIA	0 (0%)	0 (0%)	1 (1%)
Atrial-esophageal fistula	0 (0%)	0 (0%)	0 (0%)
Pericardial effusion	0 (0%)	1 (1%)	0 (0%)
Pericarditis	3 (2%)	0 (0%)	2 (2%)
Sternal wound infection	0 (0%)	0(0%)	1 (1%)
Vascular access complication	0 (0%)	1 (1%)	0 (0%)
Phrenic nerve injury	0 (0%)	2 (2%)	0 (0%)

RFCA, radiofrequency catheter ablation; CBA, cryoballoon ablation; TTA, totally thoracoscopic ablation; TIA, transient ischemic attack.

## Data Availability

Data sharing is not applicable.
